# Cryoprotectant effects of mangiferin on the quality of frozen-thawed boar sperm

**DOI:** 10.3389/fvets.2025.1706051

**Published:** 2026-01-08

**Authors:** Minhao Zhang, Zhaobo Luo, Weipu Mao, Guangyuan Zhang, Peng Gu

**Affiliations:** 1 Department of Urology, Xishan People’s Hospital of Wuxi City, Wuxi Branch of Zhongda Hospital Southeast University, Wuxi, China; 2 Department of Animal Science, College of Agriculture, Yanbian University, Yanji, Jilin, China; 3 Department of Urology, Zhongda Hospital Southeast University, Nanjing, Jiangsu, China

**Keywords:** antioxidant, boar sperm cryopreservation, mangiferin, ROS, sperm quality

## Abstract

Boar sperm is particularly vulnerable to damage at low temperatures, leading to reduced efficiency in cryopreservation. This study aimed to analyze the effect of mangiferin (Man) on boar sperm cryopreservation efficiency. Various concentrations of Man (0, 5, 10, 20, and 40 μM) were added into the cryopreservation extender and analyzed for their effects on sperm motility, kinetic parameters, acrosome integrity, plasma membrane integrity, DNA integrity, mitochondrial membrane potential, total antioxidant capacity (T-AOC) levels, hydrogen peroxide (H_2_O_2_) content, glutathione (GSH) levels, malondialdehyde (MDA) levels, and reactive oxygen species (ROS) levels. The results indicate that, in comparison to the control group, 10 μM Man significantly enhances sperm total motility, progressive motility, average path velocity, average straight-line velocity, average curvilinear velocity, and beat frequency during the cryopreservation of boar semen. Furthermore, our studies demonstrated that 10 μM Man significantly enhance mitochondrial membrane potential, acrosome integrity, plasma membrane integrity, and DNA integrity in boar sperm after thawing. Additionally, treatment with 10 μM Man markedly increase GSH levels and T-AOC in boar sperm, while simultaneously reducing MDA, H_2_O_2_, and ROS levels. Consequently, the findings of this study suggest that treatment with 10 μM Man substantially improve the efficiency of boar semen cryopreservation.

## 1 Introduction

Artificial insemination (AI) is a well-established breeding technology that has been extensively utilized in boar reproduction, primarily depending on semen preserved at 17 °C (1). However, this preservation method has several drawbacks, particularly its limited storage duration (1). In contrast, the cryopreservation of semen provides several advantages, including extended storage duration, facilitation of international trade, and improved preservation of high-quality boar genetic resources (2). This process can induce oxidative stress due to low temperatures, leading to reduced sperm motility and damage to the sperm acrosome, plasma membrane, and DNA. Consequently, these factors diminish the efficiency of semen cryopreservation and the fertilization ability of sperm post-thawing (3–6). Therefore, incorporating various antioxidants into the freezing preservation solution of boar semen represents an effective strategy to enhance the efficiency of this process.

Due to the high content of unsaturated fatty acids and low cholesterol levels in boar sperm cell membranes, these cells are particularly susceptible to oxidative stress (7). This susceptibility results in a reduced total antioxidant capacity (T-AOC) and an increased level of oxidative stress within the sperm (7). Consequently, the efficiency of cryopreservation for boar semen is lower than that of other livestock species, which restricts the utilization of frozen boar semen. During the cryopreservation of boar sperm, the intracellular antioxidant system is often insufficient to effectively neutralize the substantial amounts of reactive oxygen species (ROS) generated in the process (8). While an appropriate level of ROS is essential for the sperm capacitation, an excessive ROS can result in partial disruption of disulfide bonds in sperm proteins (9). In recent years, an increasing body of evidence has demonstrated that various antioxidants, such as astaxanthin (10), L-proline (6), spermidine (11), and MitoQ (12), significantly improve the efficiency of boar semen cryopreservation by alleviating oxidative stress.

Chinese herbal medicine is increasingly popular due to its minimal toxic side effects and strong antioxidant properties (13). Mangiferin (Man), a natural polyphenol monomer of Chinese traditional herbs with a C-glycosylxanthone structure, exhibits various biological effects, including antioxidant (14), anti-inflammatory (15), and anti-tumor activities (16) under pathological conditions. It is predominantly extracted from plants of the Man indica species (13). Numerous studies have demonstrated that Man possesses significant antioxidant properties. Huang et al. (17) demonstrated that Man can ameliorate placental oxidative stress by modulating levels of malondialdehyde (MDA), superoxide dismutase (SOD), T-AOC, and glutathione (GSH) in a preeclampsia mouse model through the activation of the PI3K/Akt/mTOR pathway. Furthermore, numerous studies have demonstrated that Man possesses a strong capacity to scavenge free radicals, thereby mitigating oxidative damage induced by ROS in various diseases, including lung injury (18), acute kidney injury (19), liver injury (20), and Parkinson’s disease (21). To date, there have been no reports on the application of Man in the cryopreservation of boar semen. Therefore, this study investigates the effects of Man on various sperm motility parameters, acrosome integrity, plasma membrane integrity, mitochondrial membrane potential, and DNA integrity during the cryopreservation of boar semen, aiming to determine its effect on sperm quality. Additionally, the study examines the activity of oxidative stress and ROS to ascertain whether Man enhances the efficiency of boar semen cryopreservation by ameliorating oxidative stress levels.

## 2 Materials and methods

### 2.1 Ethics approval

All experiments were approved by the Ethics Committee of Yanbian University (Approval Number: SYXK2020-0009). This research was conducted in strict accordance with relevant guidelines and under appropriate supervision.

### 2.2 Chemicals

The Man was procured from Shanghai Yuanye Bio-Technology Co., Ltd. (Shanghai, china). The T-AOC (A015-2-2), GSH-Px (A005-1-2), hydrogen peroxide (H_2_O_2_, A064-5-1), MDA (A003-1-2), and adenosine triphosphate (ATP, A095-1-1) were bought from Nanjing Biotechnology Co., Ltd. (Nanjing, China). All other reagents, unless specified otherwise, were sourced from Sigma-Aldrich.

The components of Cryoprotectant Extender I and II, as well as the thawing solution, were referenced from the study conducted by Álvarez-Rodríguez et al. (22). The specific components of Cryopreservation Extender I are as follows: 200 mL/L egg yolk, 194.27 mmol/L glucose, 1 million U/L streptomycin, and 102.25 mmol/L lactose. The pH value and osmotic pressure of the solution are 7.2 and 270 mOsmol/kg, respectively. The cryopreservation extender was centrifuged at 9,000 g for 10 min at 4 °C, and the supernatant was collected for storage at 4 °C. To prepare Cryoprotectant Extender II, 7% (v/v) glycerol is added to Cryoprotectant Diluent I. Additionally, Man was incorporated into Cryopreservation Extenders I and II to achieve concentrations of 0, 5, 10, 20, and 40 μM, respectively. The primary components of the thawing solution include 34.62 mmol/L ethylenediamine, 23.17 mmol/L caffeine, 2.47 mmol/L citric acid, and 138.77 mmol/L glucose, with the pH maintained at 7.4 ± 0.1.

### 2.3 Semen collection and treatment

The semen used in this experiment was collected during the spring from 12 healthy Landrace boars, each approximately 3 years old. The boars were housed in environments with optimal temperature (22 °C), humidity (70%), and natural lighting, and they had unrestricted access to feed and water. Semen was collected weekly from the boars using a hand-held method. To avoid individual differences, each sample contained a mixture of semen from randomly four boars, resulting in three distinct samples. The mixed semen was transported to the laboratory within 30 min. A total of nine mixed semen samples were collected during the formal experiment. Sperm motility parameters were analyzed using the Computer-Assisted Sperm Analysis (CASA, Songjingtianlun Biotechnology, Nanning, China) system, and only sperm samples exhibiting motility parameters exceeding 90% were utilized for subsequent experiments.

Semen cryopreservation was performed as follows: Semen samples were placed in a 15 mL centrifuge tube and centrifuged at 290 g and 37 °C for 10 min, after which the supernatant was discarded. Cryopreservation extender I was added to adjust the sperm concentration to 4 × 10^8^/mL, followed by equilibration at 17 °C for 20 min. Subsequently, Cryoprotectant II was added to further adjust the sperm concentration to 2 × 10^8^/mL, ensuring thorough mixing, and equilibrated at 4 °C for 50 min. The well-mixed semen was then transferred into 0.5 mL cryopreservation straws, with approximately 30 straws allocated per concentration treatment group. The subsequent semen cryopreservation was conducted according to the methodology established by Li et al. (23), with appropriate modifications. Freezing was carried out using a programmable freezer (CryoLogic, Melbourne, Australia) following this protocol: the temperature was decreased from 4 °C to −5 °C at a rate of −6 °C per min, maintaining this temperature for 60 s. Subsequently, the temperature was then reduced to −120 °C at the same rate and held for an additional 60 s. Upon completion of the freezing process, the straws were quickly transferred into liquid nitrogen for storage over a period of 30 days. Semen thawing was conducted as follows: rapidly remove the frozen straws from the liquid nitrogen tank and place them in a water bath at 50 °C for 10 to 15 s. After thawing, the semen must be thoroughly mixed with 300 μL of thawing solution, incubated at 37 °C, and subsequently utilized for further experiments.

### 2.4 Measurement of sperm quality and kinetic parameters

The sperm quality and kinetic parameters were analyzed with CASA system. The CASA system captures dynamic images of sperm at specific fluid layer depths using advanced microscopy and high-speed camera systems, while ensuring the viability of sperm through a constant temperature device. It records videos lasting 1–2 s at a sampling frequency of 30–60 Hz, effectively documenting the trajectories of sperm movement. The CASA system is configured to capture 30 frames per second to assess sperm quality and kinetic parameters. Additionally, all other parameters are set to motility, defined as the percentage of spermatozoa exhibiting a straightness of path (STR) greater than 75%. The minimum contrast is established at 46, the minimum cell size is set to 7 pixels, and the velocity straight line (VSL, μm/s) is required to exceed 25 μm/s. Sperm quality primarily encompasses total motility (TM), progressive motility (PM) and abnormality. Additionally, sperm kinetic parameters include VSL, average path velocity (VAP, μm/s), curvilinear velocity (VCL, μm/s), and beat-cross frequency (BCF, Hz). After thoroughly mixing the semen, take 10 μL of the sample and transferred it on a glass slide. Subsequently, place a coverslip over the sample and incubate it at 37 °C for 15 min to record the sperm TM, PM, VSL, VAP, VCL and BCF.

Sperm abnormality encompass a range of morphological defects, including the absence of tails, the presence of biflagellate bodies, excessive bending, taillessness, and head splitting. Using the CASA system to capture images of sperm, and based on the above description of sperm abnormality, approximately 200 (254 ± 40.77) sperm were selected from each sample to count the number of abnormality sperm and calculate the abnormality rate.

### 2.5 Measurement of sperm mitochondrial membrane potential

The mitochondrial membrane potential (MMP) was assessed using the JC-1 staining method, with experimental procedures adapted from the study conducted by Ma et al. (24). Initially, the JC-1 staining solutions were combined to achieve a total volume of 400 μL, resulting in a final concentration of 1 mmol/L JC-1. Subsequently, 100 μL of the semen sample was added, mixed thoroughly, and incubated in the dark at 37 °C for 30 min. A 10 μL aliquot of the mixed sample was then pipetted onto a glass slide, covered with a coverslip, and photographed using an inverted fluorescence microscope (Mshot Photoelectric Technology, Guangzhou, China) in a dark environment. Each sample is examined from five different views to ensure that the total sperm count includes a minimum of 200 (239.13 ± 25.40) sperm. Red fluorescence indicated a high MMP (hMMP), while green fluorescence represented medium and low MMP.

### 2.6 Measurement of sperm plasma membrane integrity

The integrity of the sperm plasma membrane was assessed using SYBR-14/propidium iodide (PI) staining, following the method described by Li et al. (23) with appropriate modifications. A total of 500 μL of the semen sample was centrifuged at 37 °C and 800 g for 5 min, after which the supernatant was discarded. Subsequently, HEPES buffer containing 10% BSA and 5 μL of SYBR-14 dye working solution (0.1 mmol/mL) was added and incubated in the dark at 37 °C for 5 min to stain intact sperm plasma membranes. Following this, 5 μL of PI working solution (0.1 mg/mL) was added and incubated at 37 °C for an additional 10 min to stain damaged sperm plasma membranes. Photographs were captured under a microscope in light-protected conditions, with each sample examined from five different views. This approach ensures that the total sperm count includes a minimum of 200 (233.47 ± 19.75) sperm. After staining, sperm exhibit red and green fluorescence. PI induces red fluorescence in sperm, indicating that the sperm plasma membrane is compromised. Conversely, SYBR14 causes sperm to emit green fluorescence, signifying that the sperm possess intact plasma membranes.

### 2.7 Measurement of sperm acrosome integrity

According to the research conducted by Sun et al. (25), the integrity of the sperm acrosome was assessed using peanut agglutinin (FITC-PNA) dye. Initially, a smear was prepared by placing a 30 μL semen sample on a slide, which was subsequently fixed with formaldehyde at 22–25 °C for 5–10 min. Following this, 30 μL of a PBS solution containing 100 μg/mL FITC-PNA was uniformly applied to the slide. The slide was then incubated in the dark at 37 °C for 10 min, after which it was rinsed with PBS and allowed to air dry. Next, 10 μL of antifade reagent was added to prevent fluorescence loss, and a coverslip was placed on top and sealed with colorless varnish. Finally, the integrity of the sperm acrosome was examined using a fluorescence microscope in a dark environment, ensuring that a minimum of 200 sperm (226.33 ± 18.67) were counted per sample across five views. Sperm heads with intact acrosomes exhibit complete green fluorescence, whereas those with damaged acrosomes demonstrate a loss or absence of fluorescence.

### 2.8 Measurement of sperm DNA integrity

The method for sperm DNA staining employs acridine orange (AO) staining, following specific procedures as outlined by Prochowska et al. (26). Initially, a smear is prepared using 20 μL of the semen sample, which is allowed to air dry before being fixed with formaldehyde for 10 min. Following fixation, the smear is stained with 20 μL of AO and incubated in the dark at 37 °C for 30 min. Subsequently, the sample is washed with HEPES and photographed using a fluorescence microscope in a dark environment. To ensure the reliability of the analysis, a minimum of 200 sperm (371.13 ± 27.56) are included from five different views. Sperm with intact DNA exhibits green fluorescence, whereas sperm with incomplete DNA displays red fluorescence.

### 2.9 Measurement of sperm antioxidant capacity and ATP level

The sperm antioxidant capacity test primarily encompasses T-AOC activity, GSH-Px content, H_2_O_2_ levels, and MDA levels. A total of 500 μL of the semen sample was centrifuged at 800 g and 37 °C for 5 min. After the supernatant was removed, 100 μL of RIPA lysis buffer was added. The mixture was then centrifuged at 12,000 g and 4 °C for 10 min, and the supernatant was collected for further analysis. Subsequently, the antioxidant capacity and ATP level specific detection methods were performed in accordance with the instructions provided in the reagent kit. A microplate reader (Thermo Fisher Scientific, Waltham, MA, United States) was utilized to measure specific concentrations based on the corresponding optical density (OD).

### 2.10 Measurement of sperm ROS levels

The ROS content in sperm was assessed using the DCFH-DA method, following the specific procedures outlined by Zhu et al. (27). The detailed protocol is as follows: First, 100 μL semen was centrifuged at 600 g and 4 °C for 5 min to remove the supernatant. Subsequently, 500 μL of the DCFH-DA working solution (10 μmol/L) was added, and the mixture was incubated at 37 °C in the dark for 20 min. The semen sample was then centrifuged again and washed three times with PBS. Next, the sperm was resuspended in PBS to achieve a concentration of 10^6^ sperm/mL. Subsequently, the fluorescence intensity of the sperm was measured using a flow cytometer (FACSVerse, BD Biosciences, Franklin Lakes, NJ, United States) under the green fluorescence channel at 488/510 nm, analyzing 10,000 sperm per sample.

### 2.11 Statistical analysis

All data from each experiment were assessed for normality using the one-sample Kolmogorov–Smirnov’s test. Additionally, the homogeneity of variances for all data from each experiment was evaluated using Levene’s test. All experimental results are presented as mean ± standard deviation (SD) or SEM. The data were analyzed using one-way ANOVA followed by Duncan’s multiple range test with SPSS26 (IBM, New York, NY, United States) to identify significant differences among the parameters. A *p*-value of less than 0.05 was considered statistically significant.

## 3 Results

### 3.1 Effects of various concentrations man on cryopreserved boar sperm motility

**Figure 1:**
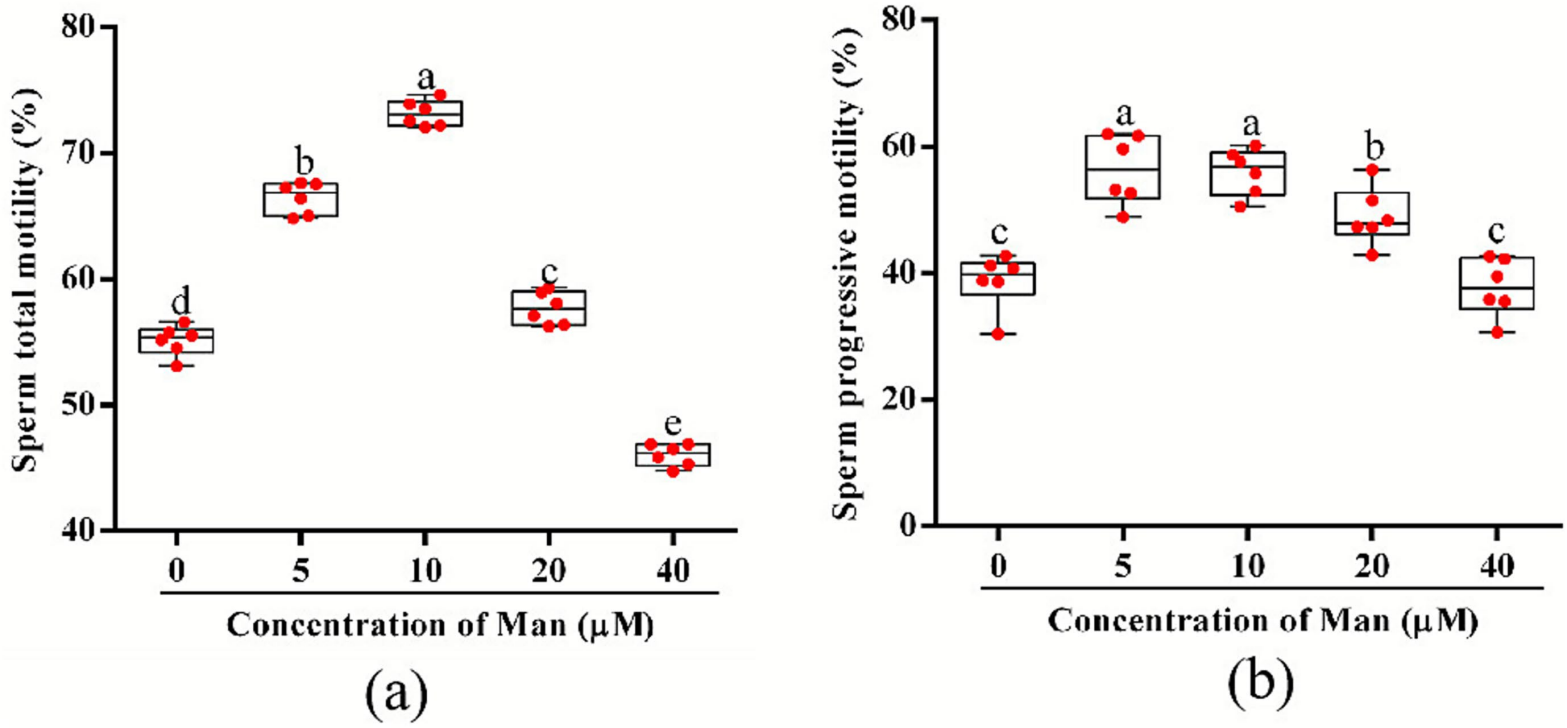
Effects of various concentrations Man on cryopreserved boar sperm TM and PM. **(a)** Sperm TM (%). **(b)** Sperm PM (%). Data are representative of six independent experiments. Different letters (a–e) indicate significant difference (*p* < 0.05). Data are means of six independent experiments (*n* = 6), with each sample consisting of three technical replicates. Results are expressed as dot plots along with means ± SEM.

The effects of various concentrations Man on the motility of cryopreserved boar sperm is shown in Figure 1. The results indicate that, compared to the control group, treatments with 5, 10, and 20 μM Man significantly enhanced sperm TM (*p* < 0.05). In contrast, the 40 μM Man treatment resulted in a significant reduction in sperm TM (*p* < 0.05). Furthermore, the 10 μM Man treatment demonstrated a significantly higher level of sperm TM compared to the other concentration (5, 20, and 40 μM) treatment groups (*p* < 0.05). Compared to the control group, treatment with 40 μM Man did not significantly affect the PM of boar sperm (*p* > 0.05). In contrast, treatments with 5, 10, and 20 μM Man significantly enhanced the PM of boar sperm (*p* < 0.05). Moreover, there were no significant differences observed between the 5 and 10 μM Man treatment groups (*p* > 0.05). Both the 5 and 10 μM Man groups exhibited significantly higher sperm PM compared to the 20 μM Man treatment group (*p* < 0.05). Additionally, the 5, 10, and 20 μM Man groups demonstrated significantly higher sperm PM than the 40 μM Man treatment group (*p* < 0.05).

**Figure 2:**
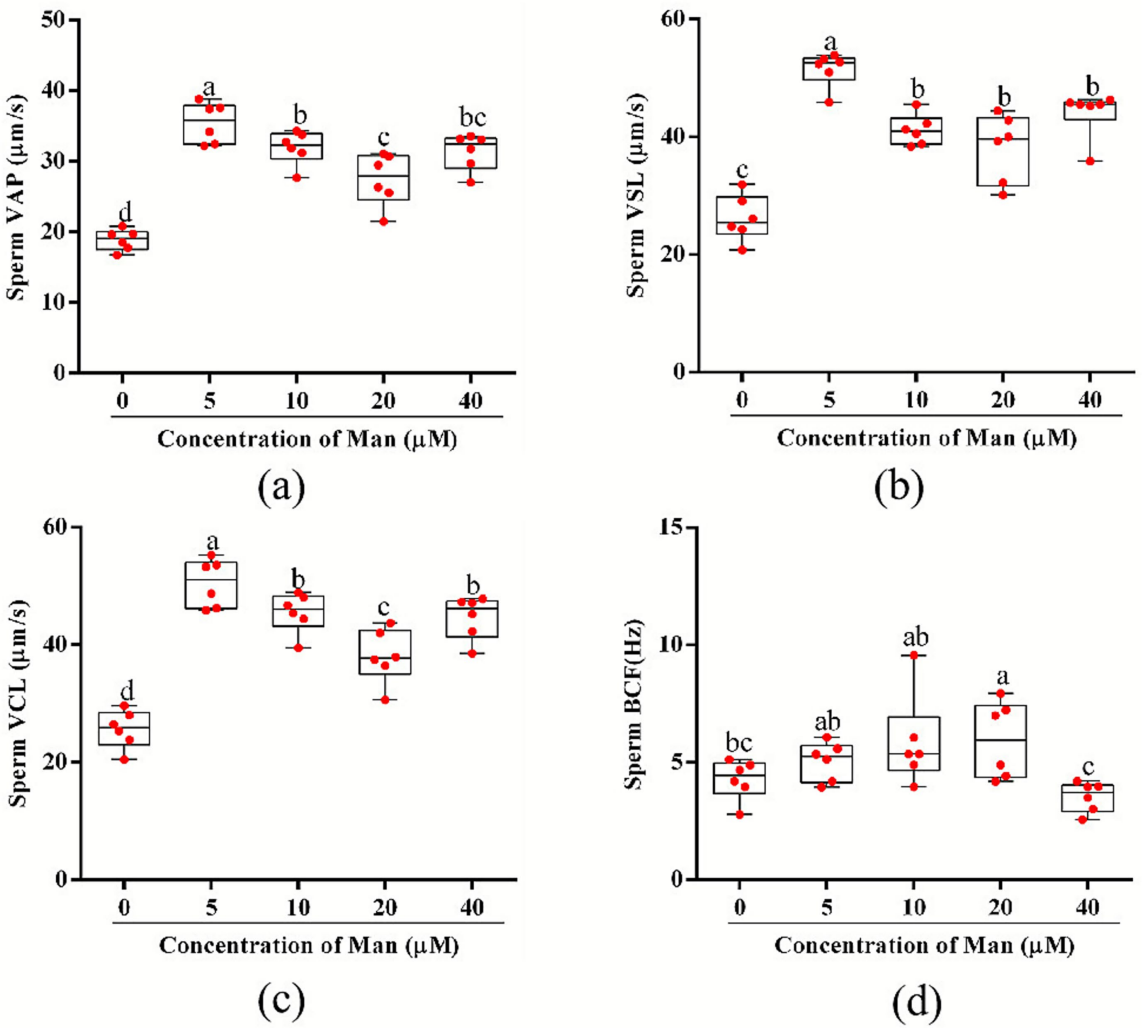
Effects of various concentrations Man on cryopreserved boar sperm kinetic parameters. **(a)** Sperm VAP (μm/s). **(b)** Sperm VSL (μm/s). **(c)** Sperm VCL (μm/s). **(d)** Sperm BCF (Hz). Data are representative of six independent experiments. Different letters (a–d) indicate significant difference (*p* < 0.05). Data are means of six independent experiments (*n* = 6), with each sample *con*sisting of three technical replicates. Results are expressed as dot plots along with means ± SEM.

The effects of varying concentrations Man on the kinetic parameters of cryopreserved boar sperm is shown in Figure 2. The results demonstrate that different concentrations of Man (5, 10, 20, and 40 μM) significantly enhanced the VAP of sperm compared to the control group (*p* < 0.05). Furthermore, treatments with 5, 10, 20, and 40 μM Man significantly increased the VSL of sperm compared to the control group (*p* < 0.05). In terms of sperm VCL, treatments with 5, 10, 20, and 40 μM Man resulted in significant increases compared to the control group (*p* < 0.05). Additionally, there was no significant difference in the BCF between the control group and the 5 or 10 μM Man treatments. However, the 20 μM Man treatment significantly improved the BCF of sperm compared to the control group (*p* < 0.05).

### 3.2 Effects of various concentrations man on cryopreserved boar sperm abnormality rate and DNA integrity

**Figure 3:**
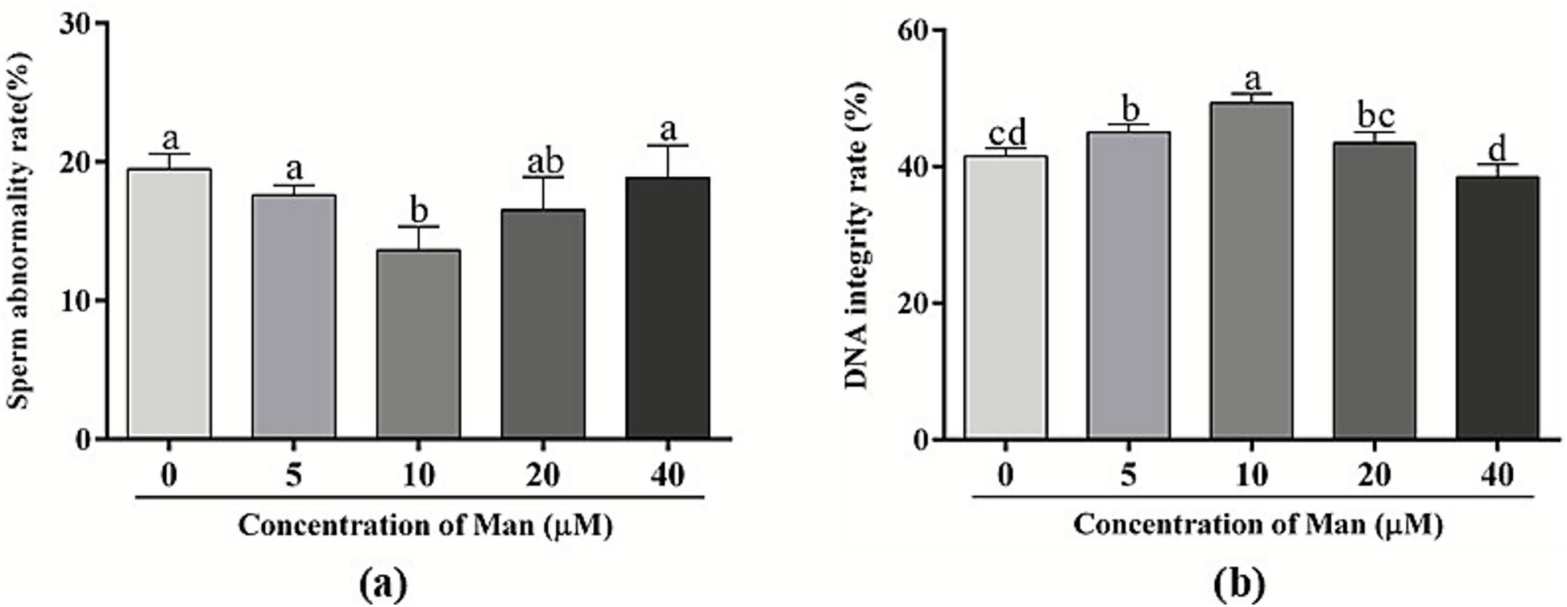
Effects of various concentrations Man on abnormality rate and DNA integrity of cryopreserved boar sperm. **(a)** Sperm abnormality rate (%). **(b)** Sperm DNA integrity rate (%). Bars represent the mean ± SD. Data are representative of three independent experiments (*n* = 3), with each sample consisting of three technical replicates. Different letters (a–c) indicate significant difference (*p* < 0.05).

The effects of various concentrations Man on the rate of sperm abnormalities in boar semen following cryopreservation is shown in Figure 3a. The results indicated that, in comparison to the control group, the treatment groups with 5, 20, and 40 μM Man did not show a significant effect on the sperm abnormality rate (*p* > 0.05). Conversely, the group treated with 10 μM of Man demonstrated a significantly lower sperm abnormality rate compared to the control group and the groups treated with 5 μM and 40 μM of Man (*p* < 0.05).

The effects of various concentrations Man on boar sperm DNA integrity following the cryopreservation of boar semen is shown in Figure 3b. The results indicate that, in comparison to the control group, treatments 5 and 10 μM Man significantly enhanced the DNA integrity of boar sperm (*p* < 0.05). Conversely, treatments with 20 and 40 μM of Man did not demonstrate a significant effect on the DNA integrity of boar sperm (*p* > 0.05). Furthermore, treatment with 10 μM Man resulted in a significantly higher DNA integrity rate in boar sperm compared to other treatment groups with different concentrations, including 5, 20, and 40 μM Man.

### 3.3 Effects of various concentrations man on cryopreserved boar sperm acrosome and plasma membrane integrity

**Figure 4:**
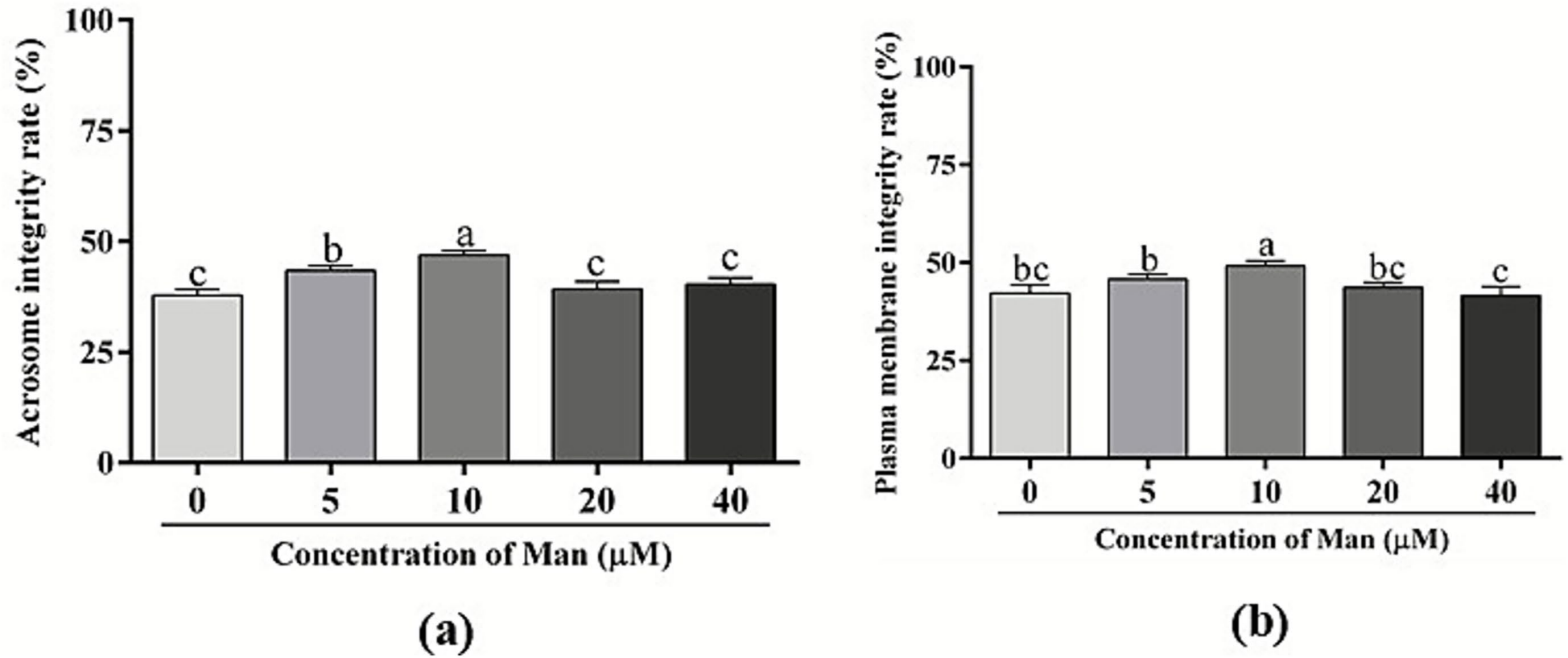
Effects of various concentrations Man on acrosome and plasma membrane integrity of cryopreserved boar sperm. **(a)** Sperm acrosome integrity (%). **(b)** Sperm plasma membrane integrity (%). Bars represent the mean ± SD. Data are representative of three independent experiments (*n* = 3), with each sample consisting of three technical replicates. Different letters (a–c) indicate significant difference (*p* < 0.05).

The effects of various concentrations Man on the acrosome integrity of cryopreserved boar sperm is shown in Figure 4a. The results demonstrate that treatments with 20 and 40 μM of Man did not significantly affect the acrosome integrity of boar sperm (*p* > 0.05). In contrast, treatment with 5 and 10 μM of Man significantly enhances the acrosome integrity of boar sperm compared to the control group (*p* < 0.05).

Furthermore, the effects of different concentrations Man on the plasma membrane integrity of cryopreserved boar sperm is shown in Figure 4b. The results indicate that treatment with 5, 20, and 40 μM Man did not significantly affect the plasma membrane integrity of boar sperm (*p* > 0.05), while 10 μM of Man significantly improves the plasma membrane integrity of boar sperm compared to the control, 5, 20, and 40 μM Man group (*p* < 0.05).

### 3.4 Effects of various concentrations man on cryopreserved boar sperm hMMP and ATP level

**Figure 5:**
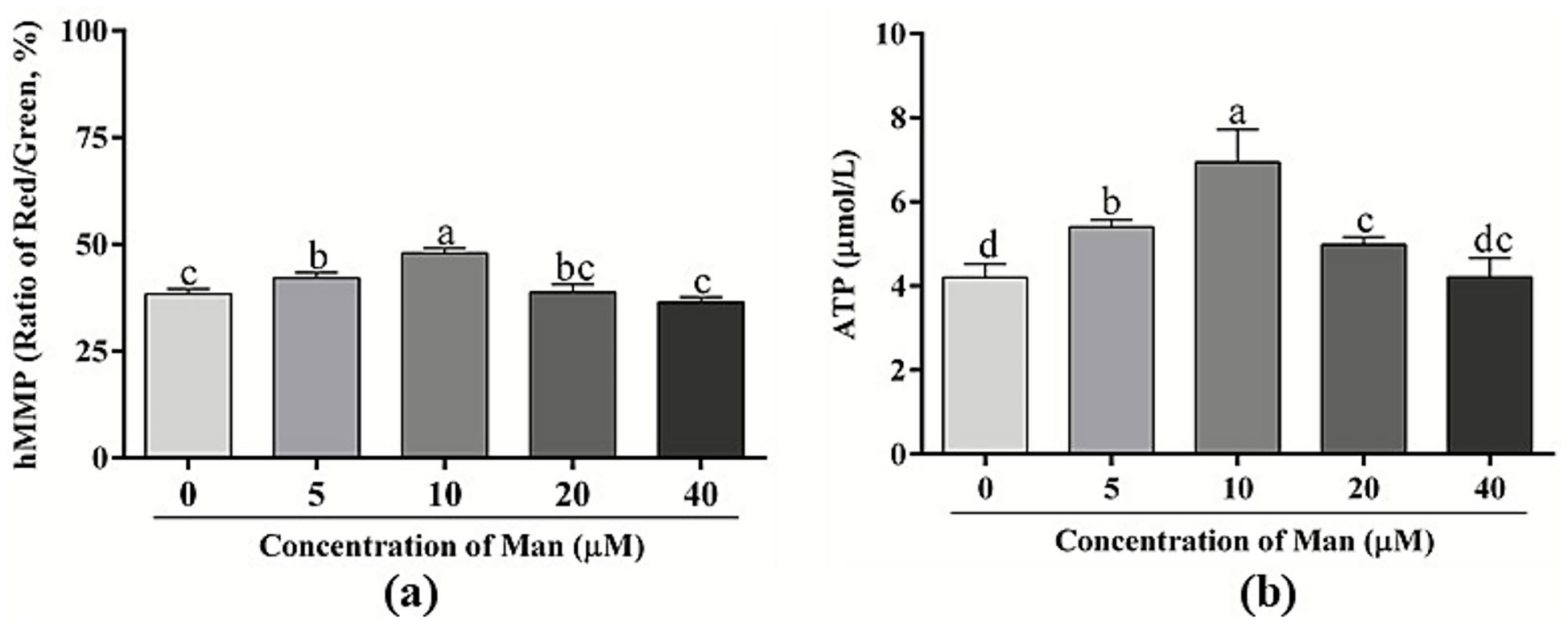
Effects of various concentrations Man on sperm hMMP and ATP level in cryopreserved boar sperm. **(a)** Sperm hMMP. **(b)** Sperm ATP level. Bars represent the mean ± SD. Data are representative of three independent experiments (*n* = 3), with each sample consisting of three technical replicates. Different letters (a–d) indicate significant difference (*p* < 0.05).

The effects of varying concentrations Man on the hMMP of cryopreserved boar sperm is shown in Figure 5a. The results indicate that treatments with 5 and 10 μM Man significantly enhanced the hMMP of boar sperm post-cryopreservation (*p* < 0.05) compared to the control group. In contrast, treatments with 20 and 40 μM Man did not significantly affect the hMMP of frozen boar sperm (*p* > 0.05).

Furthermore, the effects of different concentrations Man on the ATP level of cryopreserved boar sperm is shown in Figure 5b. Treatments with 5, 10 and 20 μM Man significantly increased ATP levels in boar sperm compared to the control group (*p* < 0.05), while 40 μM Man did not significantly influence ATP levels in boar sperm (*p* > 0.05).

### 3.5 Effects of various concentrations man on cryopreserved boar sperm antioxidant capability

**Figure 6:**
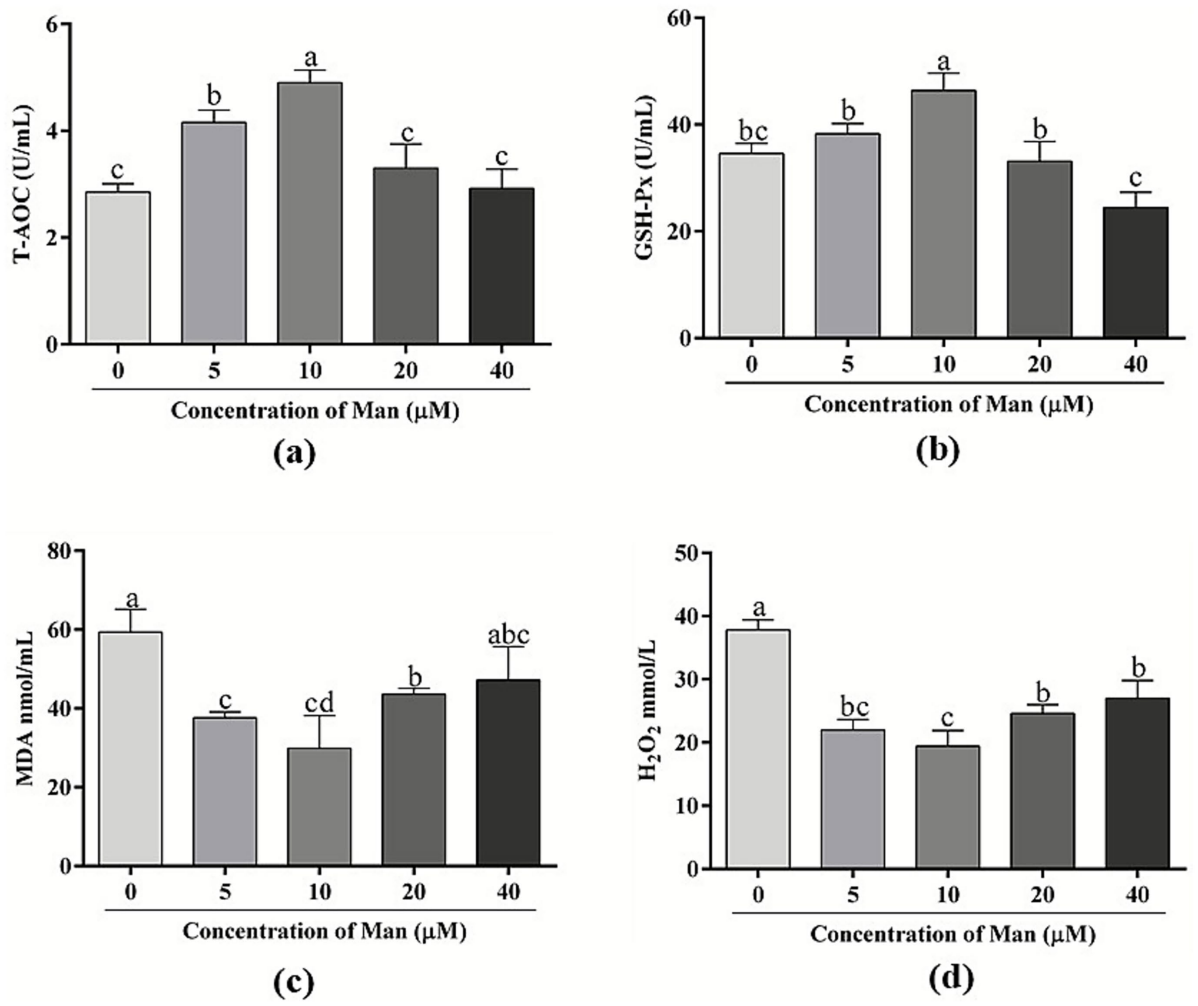
Effects of various concentrations Man on sperm antioxidant capability in cryopreserved boar sperm. **(a)** T-AOC content. **(b)** GSH content. **(c)** MDA content. **(d)** H_2_O_2_ level. Bars represent the mean ± SD. Data are representative of three independent experiments (*n* = 3), with each sample consisting of three technical replicates. Different letters (a–c) indicate significant difference (*p* < 0.05).

The effects of various concentrations of Man on the antioxidant capability of cryopreserved boar sperm is shown in Figure 6. The results indicate that treatment with 20 and 40 μM Man did not significantly affect the T-AOC levels of boar sperm compared to the control group (*p* > 0.05). In contrast, treatments with 5 and 10 μM Man significantly improved the T-AOC levels of boar sperm (*p* < 0.05). Moreover, the T-AOC levels in the 10 μM Man treatment group was significantly higher than that observed in the other treatment groups, which included the 5, 20, and 40 μM Man treatment groups (*p* < 0.05). More importantly, the GSH content in the 10 μM Man-treated group was higher than that of the control group and other concentration treatment groups (*p* < 0.05).

Furthermore, the treatment with 40 μM Man did not significantly affect the MDA content in boar sperm compared to the control group (*p* > 0.05), whereas treatments with 5, 10, and 20 μM Man significantly reduced the MDA content in boar sperm (*p* < 0.05). Additionally, treatments with 5 and 10 μM of Man demonstrated significantly lower levels of MDA compared to the 20 μM treatment group (*p* < 0.05). However, no significant difference in MDA levels was observed between the 5 and 10 μM Man treatment groups (*p* > 0.05).

Compared to the control group, the levels of H_2_O_2_ in the Man treatment groups, across various concentrations, were significantly lower (*p* < 0.05). The H_2_O_2_ level in the 10 μM Man treatment group was lower than that in the 5 μM treatment group, but the difference was not significant (*p* > 0.05). Meanwhile, the H_2_O_2_ level in the 10 μM Man treatment group was significantly lower than those in the 20 and 40 μM Man treatment groups (*p* < 0.05).

**Figure 7:**
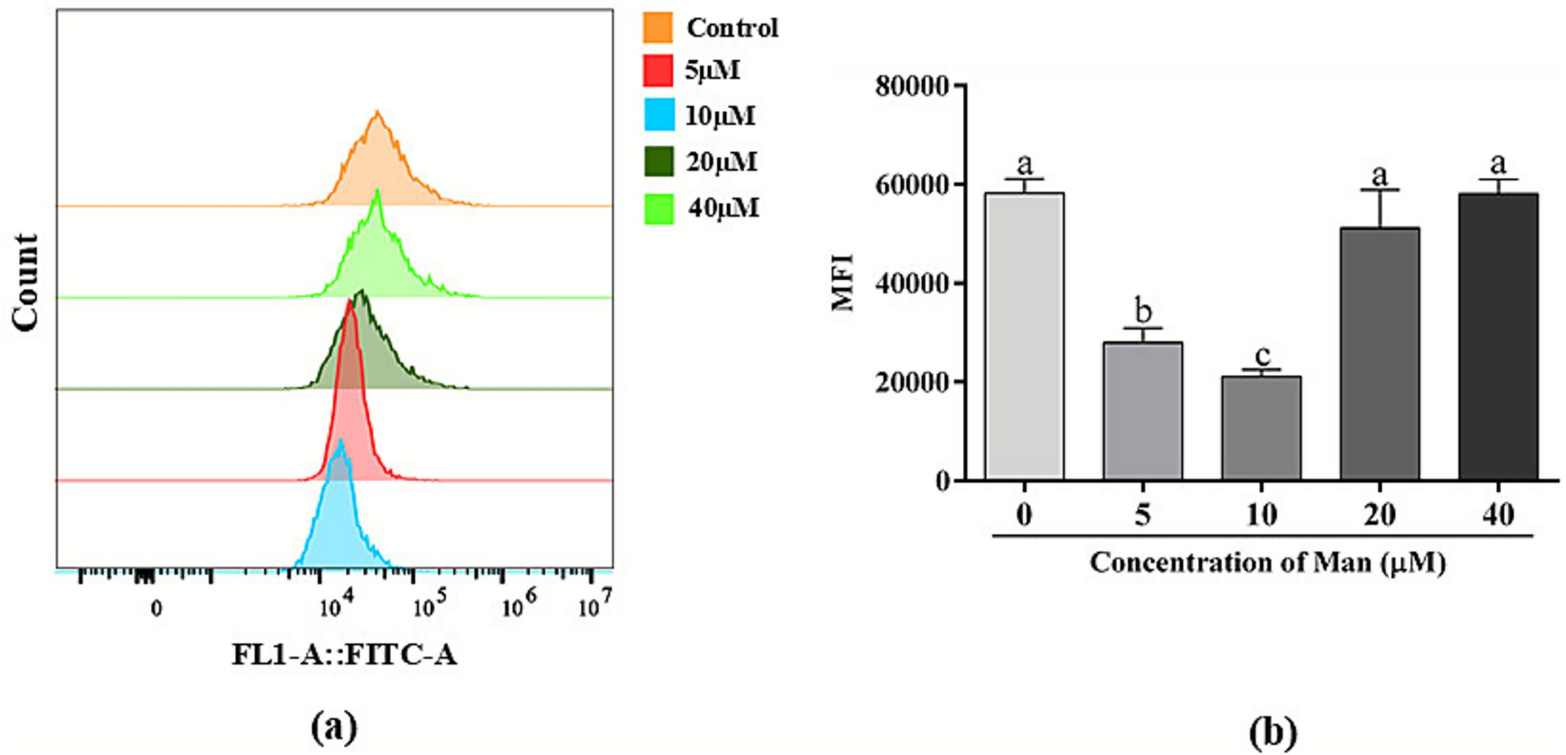
Effects of various concentrations Man on sperm ROS level in cryopreserved boar sperm. **(a)** Flow cytometry was employed to assess the ROS levels in boar sperm. **(b)** The ROS levels across different treatment groups were quantified. Bars represent the mean ± SD. Data are representative of three independent experiments (*n* = 3), with each sample consisting of three technical replicates. Different letters (a, b) indicate significant difference (*p* < 0.05).

Furthermore, the effects of different concentrations Man on the ROS content of cryopreserved boar sperm is shown in Figure 7. The results indicated that, compared to the control group, treatments with 20 and 40 μM of Man did not significantly affect the ROS content in boar sperm (*p* > 0.05). However, treatments with 10 μM of Man significantly reduced the ROS content in boar sperm compared with control and other concentration including 5, 20, and 40 μM Man groups (*p* < 0.05).

## 4 Discussion

Sperm cryopreservation has extensive applications in preserving genetic resources for humans and various male livestock (28, 29). It also plays a significant role in accelerating the improvement of breed genetic resources (29). The cytoplasmic membrane of boar sperm is rich in unsaturated fatty acids (PUFA), while the content of phospholipids and cholesterol is relatively low. This composition renders the cell membrane susceptible to oxidative stress and lipid peroxidation, resulting in low efficiency during boar semen cryopreservation (30), which limits its application in boar reproduction. Traditional Chinese medicine, characterized by minimal toxic side effects and strong antioxidant capabilities, is widely utilized in medical practices. This study demonstrated that incorporating different concentrations of the traditional Chinese medicine Man into the extender for boar semen cryopreservation significantly enhanced cryopreservation efficiency, with 10 μM Man exhibiting notable improvement.

Motility is essential for sperm progression to the ampulla of the fallopian tube (31). The motility patterns are generally classified into two categories: regular motion and hyperactivation, both of which primarily depend on the whipping motion of the tail (32). Furthermore, oxidative stress is the primary factor contributing to reduced sperm motility during the cryopreservation of boar semen (2). The findings of this study demonstrate that 10 μM Man can significantly enhance the motility and kinetic parameters of boar sperm following freeze-thawing, including TM, PM. Therefore, considering the antioxidant properties of Man, it suggests that Man may enhance sperm motility during the cryopreservation of boar semen by regulating its antioxidant capacity.

A mature sperm typically contains between 72 and 80 mitochondria (33, 34). The ATP produced by these mitochondria is essential for maintaining intracellular environmental stability and providing energy for relevant physiological processes including the acrosome reaction and egg penetration (33, 34). However, during cryopreservation, the abrupt temperature drop leads to ice crystal formation within the sperm, causing alterations in osmotic pressure and a decline in mitochondrial function (35). The results of this study indicate that 10 μM Man significantly enhances the hMMP and ATP level in boar sperm after freezing and thawing. This study indicates that Man improves mitochondrial function and ATP levels, which may facilitate the acrosome reaction and the penetration of oocytes during fertilization following subsequent thawing.

Oxidative phosphorylation in mitochondria produces by-products known as ROS, with increased ROS production occurring following mitochondrial damage (36). ROS play a dual role within organisms; at physiological concentrations, it is involved in various physiological functions in sperm, including the acrosome reaction, maturation, capacitation, and sperm-egg binding (37). However, the intracellular antioxidant system, which comprises enzymes that scavenge free radicals, is located in the cytoplasm, which contains minimal content in sperm. Research conducted by Zhu et al. (38) indicates that high levels of ROS are produced during the cryopreservation of boar semen. Excessive production of ROS leads to oxidative damage to DNA and the plasma membrane, ultimately resulting in diminished sperm motility and viability post-thawing (39). The findings of this study indicate that 10 μM of Man can significantly reduce ROS levels in thawed boar sperm, suggesting that Man may enhance the efficiency of boar semen cryopreservation by improving mitochondrial function and decreasing ROS content.

The oxidative damage induced by elevated levels of ROS is associated with impairments in cell membrane integrity, acrosome integrity, DNA stability, and mitochondrial function in sperm (40). These impairments can lead to sperm apoptosis, ultimately reducing fertility (41). In this study, we observed that 10 μM Man significantly enhanced the integrity of the sperm plasma membrane, acrosome, and DNA during the cryopreservation of boar semen. This finding suggests that Man can improve sperm quality during boar semen cryopreservation by mitigating oxidative stress.

Research indicates that MDA is a significant marker of oxidative stress and lipid peroxidation (42). This study demonstrates that treatment with 5 and 10 μM of Man significantly decreases the levels of MDA in thawed sperm. Furthermore, research across various cells types has shown that Man can significantly promote the content of GSH (21) and T-AOC (43). Consistent with these findings, we observed that treatment with 5 and 10 μM of Man significantly increases the levels of GSH and T-AOC in thawed boar sperm, thereby enhancing the antioxidant capacity of the sperm.

## 5 Conclusion

The results of this study indicate that Man enhances the efficiency of boar semen cryopreservation by reducing oxidative stress, with an optimal concentration identified at 10 μM. Therefore, it is anticipated that the findings of this study will provide a theoretical reference for the application of Man in boar semen cryopreservation. The pregnancy rate following AI serves as a critical indicator for evaluating sperm quality. Consequently, our subsequent research will concentrate on determining the applicability of the enhanced efficiency of cryopreservation of boar semen, achieved through the use of Man, within the pig farming industry.

## Data Availability

The raw data supporting the conclusions of this article will be made available by the authors, without undue reservation.
